# Nurse perceptions of practice environment, quality of care and patient safety across four hospital levels within the public health sector of South Africa

**DOI:** 10.1186/s12912-024-01992-z

**Published:** 2024-05-13

**Authors:** Immaculate Sabelile Tenza, Alwiena J. Blignaut, Suria M. Ellis, Siedine K. Coetzee

**Affiliations:** 1 School of Nursing Science, Faculty of Health Sciences, North-West University, Potchefstroom, South Africa; 2 Department of Statistical Consultation, Faculty of Humanities, North-West University, Potchefstroom, South Africa

**Keywords:** Nurse practice environment, Patient safety, Quality of care, South Africa, Public hospitals

## Abstract

Improving the practice environment, quality of care and patient safety are global health priorities. In South Africa, quality of care and patient safety are among the top goals of the National Department of Health; nevertheless, empirical data regarding the condition of the nursing practice environment, quality of care and patient safety in public hospitals is lacking.

**Aim**

This study examined nurses’ perceptions of the practice environment, quality of care and patient safety across four hospital levels (central, tertiary, provincial and district) within the public health sector of South Africa.

**Methods**

This was a cross-sectional survey design. We used multi-phase sampling to recruit all categories of nursing staff from central (*n* = 408), tertiary (*n* = 254), provincial (*n* = 401) and district (*n* = 244 [large *n* = 81; medium *n* = 83 and small *n* = 80]) public hospitals in all nine provinces of South Africa. After ethical approval, a self-reported questionnaire with subscales on the practice environment, quality of care and patient safety was administered. Data was collected from April 2021 to June 2022, with a response rate of 43.1%. ANOVA type Hierarchical Linear Modelling (HLM) was used to present the differences in nurses’ perceptions across four hospital levels.

**Results**

Nurses rated the overall practice environment as poor (M = 2.46; SD = 0.65), especially with regard to the subscales of nurse participation in hospital affairs (M = 2.22; SD = 0.76), staffing and resource adequacy (M = 2.23; SD = 0.80), and nurse leadership, management, and support of nurses (M = 2.39; SD = 0.81). One-fifth (19.59%; *n* = 248) of nurses rated the overall grade of patient safety in their units as poor or failing, and more than one third (38.45%; *n* = 486) reported that the quality of care delivered to patient was fair or poor. Statistical and practical significant results indicated that central hospitals most often presented more positive perceptions of the practice environment, quality of care and patient safety, while small district hospitals often presented the most negative. The practice environment was most highly correlated with quality of care and patient safety outcomes.

**Conclusion**

There is a need to strengthen compliance with existing policies that enhance quality of care and patient safety. This includes the need to create positive practice environments in all public hospitals, but with an increased focus on smaller hospital settings.

## Background

Improving the nurse practice environment, quality of healthcare and patient safety has become a global priority [1]. This is because countries worldwide are striving to provide universal health coverage (UHC) to their citizens, and quality and safe care has been prioritised in the agenda to achieve UHC [2, 3]. Recently there has been an increase in scholarly attention on the relationship between the nurse practice environment, quality of healthcare and patient safety, with global consensus that a positive nurse practice environment contributes positively to these [4].

The nurse practice environment is defined as the organisational characteristics of a work context that facilitate or constrain professional nursing practice [5]. Quality of care is the degree to which health services for individuals and populations increase the likelihood of the desired health outcomes [6], and patient safety is a dimension of quality of care and is defined as the avoidance of unintended or unexpected harm to people during the provision of healthcare [1].

Studies on the nurse practice environment have focused on nurse participation in organisational affairs, staffing and resource adequacy, and nurse leadership, management, and support of nurses, nurse-physician collegial relations, and foundations of quality of care [7–9]. A recent meta-analysis [10] found consistent and significant associations between the practice environment and quality of care and patient safety, based on data from 1,368,420 patients in 22 countries (including South Africa), 141 nursing units, 165,024 nurses, and 2677 hospitals. Ten years ago, a South African article—the only one from Africa included in this meta-analysis—showed the following trends: 52.3% of nurses assessed their practice environment as either poor or fair, 20.7% rated the quality of care as either poor or fair, and 5.5% rated patient safety as inadequate or failing [11]. In all cases, the public sector had worse outcomes than the private sector; and the study concluded that the nurse practice environment was significantly associated with better nurse and patient outcomes [11]. No national study has since followed this, with most studies focusing on small-scale or single-site qualitative and quantitative descriptive studies. Furthermore the variables of interest were explored separately from each other, such as the influence of the nurse practice environment on nurse outcomes [12, 13], professional nurses’ understanding of quality nursing care [14], with a primary focus on patient safety culture [13, 15–17]. Quality of care and patient safety studies in South Africa reported negative experiences of health providers, but these were not linked with the practice environment, even with ample evidence of its influence. One significant issue is the existence of policy documents that govern quality of care and patient safety in the nation. These include the following: the Patient Rights Charter, the Batho Pele principles, the National Core Standards framework [18], the National Guideline for Patient Safety Incident Reporting [19], and the Ideal Facility Framework [20]. Despite the aforementioned governmental obligations, achieving quality in healthcare continues to be a struggle [21]. This has been evidenced by the reports of litigations experienced by public health hospitals [22]. A major concern of the National Department of Health is the sudden increase in expenditure related to medico-legal claims. In the 2020/2021 financial year, more than ZAR6.5 billion (US $343,496.02) was awarded in medicolegal claims in the public sector [23].

Nurses as frontline, street-level bureaucrats in the implementation of the policies related to quality of care and patient safety in healthcare have critical experience of the nurse practice environment, quality of care and patient safety, and their views could contribute to future improvements [5]. Given existing evidence that the nurse practice environment influences quality of care and patient safety, it is important to understand the current situation. While there are existing policies directing quality of care and patient safety, it is not known how having these policies in place shapes the nurse practice environment, perceived quality of care and patient safety. This article expands on the findings of a previous national study [11], which demonstrated that the public sector had a more negative nurse practice environment, quality of care and patient safety. To add to the body of knowledge, this study examines the public sector and four hospital levels: central, tertiary, provincial, and district (small, medium, and large) hospitals. Hence this national study sought to examine nurses' perceptions of the practice environment, quality of care and patient safety across four hospital levels within the public health sector of South Africa.

## Methods

### Theoretical framework

This study is based on the theoretical framework of Tvedt et al. [24], which is a system perspective based on the model of Donabedian and modified by Battles (2006) to show how hospital structures and practice environment features improve quality of care and patient safety [24]. These outcomes are specifically identified as quality of care, patient safety (work-related outcome measures), and low-frequency adverse events and self-care ability (patient-related outcome measures).

### Study context

This study was conducted in all nine provinces of South Africa, namely, Northern Cape, Western Cape, Eastern Cape, Free State, North West, Gauteng, Limpopo, Mpumalanga, and KwaZulu-Natal. South Africa has a two-tier healthcare system, with a public and a private sector [18, 25]. The public sector is state-funded and caters to the majority – 71% – of the population [19, 25]. The private sector is largely funded through individual contributions to medical aid schemes or health insurance, and serves a minority of the population [20, 25]. This study focused on the public sector hospitals as they cater for the majority of the population. There are five categories of hospitals in the public sector, including district, regional, tertiary, central, and specialised hospitals, which are categorised according to the nature and extent of services provided and size [26]. The first point of entry to the South African health system is through primary healthcare (PHC) facilities, often referred to as clinics. Patients are referred from PHC facilities to district hospitals, regional, tertiary and central hospitals or specialised hospitals [26]. District hospitals are categorised into small, medium, and large district hospitals. Small district hospitals have between 50 and 150 beds; medium district hospitals have between 150 and 300 beds; and large district hospitals have between 300 and 600 beds [26]. These hospitals serve a defined population within a health district and support PHC facilities, providing services that include in-patient, ambulatory health services as well as emergency health services [26]. A regional hospital has between 200 and 800 beds and receives referrals from several district hospitals. Regional hospitals provide health services on a 24-h basis to a defined regional population, limited to provincial boundaries [26]. A tertiary hospital has between 400 and 800 beds and receives referrals from regional hospitals not limited to provincial boundaries, and also provides specialist level services [26]. A central hospital has a maximum of 1200 beds, receives patients referred from more than one province, and provides tertiary hospital services; they may also provide national referral services, including conducting research. A central hospital is attached to a medical school as the main teaching platform [26].

### Study design

This study had a cross-sectional descriptive design. The STROBE checklist of items that should be included in reports of cross-sectional studies was used to guide the study and the reporting thereof.

### Population and sampling

Multi-phase sampling was applied in the public sector. Purposive sampling was applied to the selection of hospitals in the public sector. A total of 27 hospitals were included by selecting the largest central or tertiary hospital in every province, and the provincial and district hospital in closest proximity to the selected central or tertiary hospital. The district hospitals were further stratified into large (*n* = 2), medium (*n* = 3), and small (*n* = 4) hospitals. Specialist hospitals were excluded. All in-patient medical and surgical units were included. Total population sampling was applied to all categories of nursing staff (registered nurses, community service nurses, enrolled nurses [2-year diploma], and enrolled nursing auxiliaries [1-year certificate]), including temporary staff, in these selected units. Nurses had to have worked in the respective unit for at least three months, and student nurses were excluded. The total sample of participants was as follows: central *n* = 408; tertiary, *n* = 254; provincial, *n* = 401; and district, *n* = 244 [large *n* = 81; medium *n* = 83 and small *n* = 80]). Data were collected from April 2021 to June 2022. A sample size calculation was performed in g-power using the F-tests as the Test Family and the ANOVA: Fixed effects, special, main effects and interactions as the Statistical test in order to take the structure of the data into account. The parameters were specified as follow: Effect size f as and large (0.4) and medium (0.25), α err prob as 0.05, Power (1-β err prob) as 0.95, Numerator df as 10, Number of groups 6. The total sample sizes calculated were 162 and 400, which is well below the realised sample size of 1307. Total population sampling was used and not a random sample, thus no generalisations are made beyond the study population of nurses from these hospitals.

### Instruments

In accordance with the theoretical framework of Tvedt et al., the variables measured included practice environment, quality of care, self-care ability, patient safety, and adverse events [24]*.* The practice environment was measured using the Practice Environment Scale of the Nurse Work Index Revised (PES-NWI-R). It consists of 32 questions and is divided into five subscales measuring nurse participation in hospital affairs; nursing foundations for quality of care; nurse manager ability, leadership, and support of nurses; staffing and resource adequacy; and collegial nurse-physician relations. The questions are measured on a Likert scale from 1 to 4, where 1 represents strongly disagree and 4 strongly agree. A mean score of 2.5 or more is indicative of a positive practice environment. This tool was found to be valid and reliable in many countries, including South Africa [27].

Quality of care was measured using the following question: In general, how would you describe the quality of nursing care delivered to patients on your unit/ward? The question was measured on a Likert scale from 1 to 5, where 1 represented excellent and 5 poor. Self-care ability was measured using one question (How confident are you that your patients and their caregivers can manage their care after discharge?), measured on a Likert scale from 1 to 4, where 1 represented very confident and 5 not at all confident.

Patient safety was measured using the following question: Please give your current practice setting an overall grade on patient safety. This was measured on a Likert scale from 1 to 5, where 1 represented excellent and 5 represented failing. The other eight items came from the Hospital Survey on Patient Safety Culture (HSOPSC) [28]. They were answered on a Likert scale from 1 to 5, where one represented strongly agree and five strongly disagree.

Finally, adverse events were measured by five questions on a five-point scale, where 1 represented never and 5 represented daily. These questions have been employed in multi-country research in South Africa [29], Europe [30], the United States of America [31], and Asia [32]. The specific outcomes have also been used in a meta-analysis [33]. The authors tried to control for response bias and subjectivity by asking neutrally worded questions, using anonymous surveys, ensuring that answer options were not leading, and that the order of the answers was randomised. i.e. the range for the practice environment was 1 = Strongly disagree.

4 = Strongly agree (ascending order), while quality of care and patient safety ranged from 1 = Excellent; 4 = Poor (descending order).

### Data collection

Data collection took place between April 2021 and June 2022 after ethics approval and obtaining permission from relevant health departments. A team of trained field workers visited the hospitals to administer a paper-based survey to all of the consenting nurses in the hospitals, according to participation criteria. Upon arrival at each hospital, each unit manager was approached and a discussion was held between researcher, manager and staff regarding permission to do a survey among nurses in the unit. The discussion gave detailed information about the study, including the voluntary nature of participation, with an invitation to participate. The survey forms were given to the participants and they were allowed to complete them at a time convenient to them. The survey was completed anonymously, and participants were requested to return them in a sealed envelope via a sealed box with a post-box split, which was placed in all departments in the participating hospitals. The contents of these boxes were emptied by the researcher at the end of each day and removed a week later upon completion of data collection at the selected hospital.

### Quantitative data analysis

Data was analysed using SPSS [34]. Descriptive statistics were used to analyse the demographic data, and data from each subscale representing the practice environment, quality of care and patient safety. These described frequencies, percentages, means and standard deviations. ANOVA type Hierarchical Linear Modelling (HLM), with *p*-values for all effects and interactions were calculated to present the differences in nurses’ perceptions of the practice environment, quality of care and patient safety across four hospital levels within the public health sector of South Africa, as the means of the different hospital levels and not the regression coefficients were important in the interpretation of the results. After the ANOVA type HLM, pair wise post-hoc comparisons were done to determine the statistically significant differences between the groups. Additionally, effect sizes were computed to determine which of these differences were important in practice. Where significant *p*- values lead to generalisations of results, effect sizes only indicate whether the differences in the sample groups were important in practice and are not used for generalisation if the *p*-values are not significant. Effect sizes were calculated and the magnitude of difference between the groups indicated as 0.2 = small, 0.5 = medium, 0.8 = large. Correlations between aspects of the nurse practice environment, quality of care and patient safety were also explored for the entire sample with 0.1. = small; 0.3 = medium and 0.5 = large relationships. Normality of the data was tested using the Kolmogorov–Smirnov test, but due to the unlikelihood of non-significant *p*-values in such a large sample size, more significance was ascribed to results from Q-Q plots. The points in the Q-Q plot lies close enough to the straight line to retain the assumption that the data distribution is normal for all variables [35].

## Results

### Demographic data

We obtained a 43.1% response rate. As indicated in Table 1, the majority of the participants were female (*n* = 1159; 88.7%), working on a full-time basis (*n* = 1158; 89.35%) and in the registered nurse/midwifery category (*n* = 593; 45.58%). Most nurses worked in the surgical units (*n* = 483; 36.95%), and we received most participation from the central level hospitals (*n* = 408; 31.22%).

**Table 1:** Demographic data Item

Variable	Options	Frequency (%)
Sex	Female Male	**1159 (88.74)** 147 (11.26)
Employment status	Full-time Part-time Agency	**1158 (89.35)** 36 (2.78) 102 (7.87)
Nursing category	RN/midwife CSN EN ENA	**593 (45.58)** 36 (2.77) 296 (22.75) 376 (28.90)
Specialty of current unit	Medical Surgical Paediatric Oncology Burns COVID-19	467 (35.73) **483 (36.95)** 270 (20.66) 11 (0.84) 9 (0.69) 67 (5.13)
Hospital level	Central Tertiary Provincial District (large) District (medium) District (small)	**408 (31.22** 254 (19.43) 401 (30.68) 81 (6.20) 83 (6.35) 80 (6.12)

*RN* Registered nurse, *CSN* Community service nurse, *EN* Enrolled nurse, *ENA* Enrolled nursing assistant

#### Nurse practice environment

The overall practice environment was not considered to be positive (M = 2.46; SD = 0.65), especially with regard to the subscales of nurse participation in hospital affairs (M = 2.22, SD = 0.76), staffing and resource adequacy (M = 2.23; SD = 0.80), and nurse manager ability, leadership, and support of nurses (M = 2.39; SD = 0.81), see Table 2.

**Table 2:** Subscales of the nurse practice environment

**Subscales**	**Scale range**	**Mean (SD)**
Nurse participation in hospital affairs	1 = Strongly disagree	2.22 (0.76)
Nurse foundations of quality of care	4 = Strongly agree	2.79 (0.65)
Nurse manager ability, leadership, and support of nurses		2.39 (0.81)
Staffing and resource adequacy		2.23 (0.80)
Collegial nurse-physician relationship		2.67 (0.81)
Total: Practice environment		2.46 (0.65)

Table 3 provides an overview of responses to items on quality of care, patient safety, and adverse events.

**Table 3:** Responses related to items on quality of care, patient safety and adverse events

Items	Scale range	Scored negatively % (f)	Mean (SD)
**Quality of care**			
In general how would you describe the **quality of nursing care delivered to patients** in your work setting?	1 = Excellent; 4 = Poor	38.45 (486)	2.26 (0.87)
How confident are you that **your patients and their caregivers can manage their care after discharge**?	1 = Very Confident; 4 = Not at all Confident	52.22 (658)	2.47 (0.89)
**Patient safety**			
Please give your current practice setting an **overall grade on patient safety**	1 = Excellent; 5 = Failing	19.59 (248)	2.52(1.12)
AHRQ—**Relies** too much on **temporary, float or agency staff**	1 = Strongly Agree; 5 = Strongly Disagree	35.95 (430)	3.20 (1.42)
AHRQ—Regularly **review work processes** to determine if changes are needed to improve patient safety		27.35 (320)	2.60 (1.26)
AHRQ—**Staff speak up** if they see something that may negatively affect patient care		12.58 (152)	2.02 (1.08)
AHRQ—Staff feel like their **mistakes are held against them**		64.38 (770)	2.37 (1.25)
AHRQ—There is a **lack of support for staff** involved in patient safety errors		63.15 (749)	2.44 (1.28)
AHRQ—We discuss ways to **prevent errors** from happening again		12.87 (151)	2.08 (1.09)
AHRQ—Staff feel **free to question the decisions** or actions of those in authority		42.22 (505)	3.02 (1.35)
AHRQ—The actions of hospital management show that patient **safety is a top priority**		27.97 (339)	2.53 (1.37)
**Adverse events**			
		**Scored weekly and daily % (f)**	
**Medication errors**	1 = Never; 5 = Daily	7.77 (93)	1.80 (1.03)
**Patient falls**		2.42 (29)	1.57 (0.73)
**Hospital acquired infection**		9.19 (108)	2.07 (1.05)
**Unintended harm** to patients		4.54 (53)	1.66 (0.91)
**Complaints** from patients or their families		21.32 (252)	2.65 (1.21)

#### Quality of care

When asked about their perception of the quality of nursing care delivered to patients in their work setting, a third of participants (38.45%; *n* = 486) indicated a negative outcome, and more than half of the nurses reported that they lacked confidence in patient or caregiver post-discharge care abilities (52.22%; *n* = 658).

#### Patient safety

As indicated in Table 3, the overall grade for patient safety was rated as poor or failing by 19.59% (*n* = 248) of participants, and 430 participants (35.95%) agreed that there was a high reliance on temporary staff in their hospitals. In addition, more than half of the participants strongly agreed that their mistakes were held against them (64.38%; *n* = 770), and that there was a lack of support for staff involved in patient safety errors (63.15%; *n* = 749). Close to half felt that they could not question the decisions or actions of those in authority when related to patient safety issues (42.22%; *n* = 505).

#### Adverse events

The subscale on adverse events examined the weekly and daily occurrence of adverse events. At least 21.32% (*n* = 252) of the participants experienced complaints weekly or daily, while 9.29% (*n* = 108) reported a weekly or daily incidence of hospital-acquired infections, and 7.77% (*n* = 93) weekly or daily medication errors.

Table 4 shows several effect sizes between the different levels of hospitals; however, only medium effect sizes will be reported on. Regarding the practice environment, there were medium practical effects between central hospitals and the small district hospitals for nurse participation in hospital affairs (*r* = 0.40; *p* = 0.291), nursing foundations for quality of care (*r* = 0.44; *p* = 0.469), and nurse manager ability, leadership, and support of nurses (*r* = 0.45; *p* = 0.484), where central hospitals reported a more positive perception of these elements. There were also medium practical effects between provincial hospitals and the small district hospitals for nurse participation in hospital affairs (*r* = 0.40; *p* = 0.211) and nursing foundations for quality of care (*r* = 0.43; *p* = 0.398), where provincial hospitals reported a more positive perception of these elements of the practice environment.

**Table 4:**
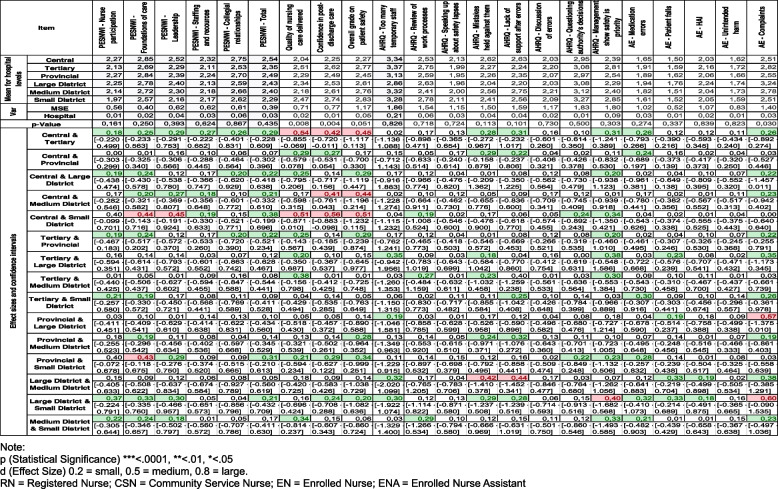
Effect sizes between the different levels of hospitals on nurse practice environment, quality of care and patient safety

Regarding the quality of care, there were medium practical effects with statistical significance between central hospitals and tertiary hospitals (*r* = 0.54; *p* = 0.015) and small district hospitals (*r* = 0.51; *p* = 0.061), where central hospitals reported better quality of care. Regarding patients’ self-care ability, there were medium practical effects between central hospitals and tertiary hospitals (*r* = 0.42; *p* = 0.042) as well as medium district hospitals (*r* = 0.41; *p* = 0.110) and small district hospitals (*r* = 0.56; *p* = 0.007), where central hospitals reported more confidence in patients’ ability to manage their own care after discharge.

Regarding patient safety, there were medium practical effects between central hospitals and tertiary hospitals (*r* = 0.45; *p* = 0.178), and also between medium district hospitals (*r* = 0.44; *p* = 0.399), and small district hospitals (*r* = 0.51; *p* = 0.178), where central hospitals reported higher grades of patient safety. Regarding staff feeling that their mistakes are held against them, there was a medium practical effect between small and medium district hospitals (*r* = 0.42; *p* = 0.681), where small district hospitals reported that mistakes were held against them more often. There was also a medium practical effect between medium and large district hospitals regarding lack of support for staff involved in patient safety errors (*r* = 0.44; *p* = 0.572), where small district hospitals reported less support for staff involved in patient safety errors. Finally, there was a medium practical effect between large and small district hospitals regarding the actions of hospital management showing that patient safety is a top priority (*r* = 0.40; *p* = 0.856), where small district hospitals felt that the actions of hospital management showed that patient safety is a top priority.

Complaints were the only adverse event that had a medium practical effect, these effects being between provincial hospitals and large district hospitals (*r* = 0.57; *p* = 0.056), and large district hospitals and small district hospitals (*r* = 0.60; *p* = 0.114), where large district hospitals had a greater incidence of complaints.

As shown in Table 5, all practice environment subscales showed medium to large negative correlations with the quality of nursing care delivered (*r* = -3.20 to *r* = -4.28; *p* = 0.00) and that patients and their caregivers can manage care after discharge (*r* = -0.282 to *r* = -0.327; *p* = 0.00). When considering the correlations of the practice environment on overall grade of patient safety, the practice environment had a large negative correlation (*r* = -0.405; *p* = 0.00), especially regarding nurse foundations of quality of care (*r* = -0.411; *p* = 0.00). Furthermore, medium negative correlations were noted between overall grade of patient safety and staffing and resources (*r* = -0.347; *p* = 0.00) and nurse management, leadership, and support of nurses (*r* = -0.340; *p* = 0.00), nurse participation (*r* = -0.323; *p* = 0.00) and collegial nurse-physician relationships (*r* = -0.299; *p* = 0.00). This shows that the more that participants agreed with positive statements about the nurse practice environment, the better they rated their quality of care, the more confidence they had in their patients’ post-discharge management, and the better they rated their overall grade on patient safety.

**Table 5:** Correlation matrix for nurse practice environment, quality of care and patient safety

	PESNWI Nurse Participation	PESNWI Foundations of Quality of Care	PESNWI Leadership	PESNWI Staffing resources	PESNWI Collegial Nurse Physician Relationships	PESNWI Total	Please give your current practice setting an overall grade on patient safety	In general how would you describe the quality of nursing care delivered to patients in your work setting?	How confident are you that your patients and their caregivers can manage their care after discharge?	AHRQ—Relies too much on temporary, float or agency staff	AHRQ—Regularly review work processes to determine if changes are needed to improve patient safety	AHRQ—Staff speak up if they see something that may negatively affect patient care	AHRQ—Staff feel like their mistakes are held against them	AHRQ—There is a lack of support for staff involved in patient safety errors	AHRQ—We discuss ways to prevent errors from happening again	AHRQ—Staff feel free to question the decisions or actions of those in authority	AHRQ—The actions of hospital management show that patient safety is a top priority
In general how would you describe the quality of nursing care delivered to patients in your work setting?	-0.320**	-0.428**	-0.345**	-0.356**	-0.335**	-0.421**	0.563**										
How confident are you that your patients and their caregivers can manage their care after discharge?	-0.287**	-0.327**	-0.282**	-0.301**	-0.286**	-0.352**	0.357**	0.438**									
Please give your current practice setting an overall grade on patient safety	-0.323**	-0.411**	-0.340**	-0.347**	-0.299**	-0.405**											
AHRQ—Relies too much on temporary, float or agency staff	0.006	0.081*	0.003	0.035	0.031	0.035	-0.039	-0.053	-0.016								
AHRQ—Regularly review work processes to determine if changes are needed to improve patient safety	-0.244**	-0.275**	-0.233**	-0.221**	-0.185**	-0.273**	0.258**	0.273**	0.166**								
AHRQ—Staff speak up if they see something that may negatively affect patient care	-0.136**	-0.226**	-0.111*	-0.089*	-0.180**	-0.173**	0.169**	0.209**	0.129**								
AHRQ—Staff feel like their mistakes are held against them	0.235**	0.184**	0.278**	0.190**	0.200**	0.259**	-0.144**	-0.176**	-0.159**								
AHRQ—There is a lack of support for staff involved in patient safety errors	0.278**	0.266**	0.302**	0.239**	0.247**	0.315**	-0.167**	-0.156**	-0.160**								
AHRQ—We discuss ways to prevent errors from happening again	-0.179**	-0.222**	-0.223**	-0.140**	-0.168**	-0.219**	0.170**	0.191**	0.080*								
AHRQ—Staff feel free to question the decisions or actions of those in authority	-0.313**	-0.220**	-0.314**	-0.233**	-0.198**	-0.304**	0.222**	0.210**	0.174**								
AHRQ—The actions of hospital management show that patient safety is a top priority	-0.333**	-0.362**	-0.348**	-0.259**	-0.222**	-0.358**	0.372**	0.305**	0.216**								
AE Medication errors	-0.049	-0.171**	-0.101*	-0.097*	-0.157**	-0.135**	0.208**	0.230**	0.149**	-0.033	0.153**	0.122*	-0.049	-0.056	0.160**	0.070*	0.114*
AE Patient falls	-0.042	-0.121*	-0.065*	-0.111*	-0.144**	-0.114*	0.223**	0.237**	0.202**	-0.044	0.143**	0.125**	-0.029	-0.058	0.180**	0.100*	0.162**
AE HAI	-0.069	-0.127**	-0.090*	-0.137**	-0.162**	-0.140**	0.166**	0.173**	0.206**	**-0.143**	0.059	0.085*	-0.054	-0.027	0.083*	0.130**	0.147**
AE Unintended harm	-0.074*	-0.157**	-0.102*	-0.073*	-0.148**	-0.130**	0.127**	0.171**	0.136**	-0.047	0.111*	0.130**	-0.052	-0.035	0.137**	0.038	0.172**
AE Complaints	-0.118**	-0.144**	-0.138**	-0.148**	-0.193**	-0.178**	0.197**	0.249**	0.211**	0.004	0.123**	0.054	-0.118**	-0.066*	0.127*	0.078*	0.136**

* Correlation is significant at the 0.05 level

** Correlation is significant at the 0.01 level

All practice environment items, except for collegial nurse-physician relationships, had medium negative correlations with the AHRQ item that the unit regularly reviews work processes to determine if changes are needed to improve patient safety (*r* = -0.221 to *r* = -0.275; *p* = 0.00). Furthermore, foundations of quality of care showed a medium negative correlation with staff speaking up when they see something that may negatively impact patient care (*r* = -0.226; *p* = 0.00). Nurse participation (*r* = 0.235; *p* = 0.00), leadership (*r* = 0.278; *p* = 0.00), collegial nurse- physician relationship (*r* = 0.200; *p* = 0.00), and the total practice environment scale (*r* = 0.259; *p* = 0.00) all showed medium positive correlations with the AHRQ item ‘Staff feel like their mistakes are held against them’. All practice environment subscales exhibited medium correlations with the lack of support for staff involved in patient safety errors (*r* = 0.239 to *r* = 0.315; *p* = 0.00). Foundations of quality of care (*r* = -0.222; *p* = 0.00), leadership, management, and support of nurses (*r* = -0.223; *p* = 0.00), and the overall practice environment scale (*r* = -0.219; *p* = 0.00) had negative medium correlations with discussing ways to prevent errors from happening again. All except the collegial nurse-physician relationship subscale of the practice environment showed medium negative correlations with staff feeling free to question the decisions or actions of those in authority (*r* = -0.222 to *r* = -0.314; *p* = 0.00). All practice environment subscales had medium correlations with the actions of hospital staff showing that patient safety is a top priority (*r* = -0.222 to *r* = -0.362; *p* = 0.00). To explain, the more that nurses agreed with positive practice environment items, the more they would agree to positive patient safety (AHRQ) items and the more they would disagree with negative patient safety (AHRQ) items.

Overall patient safety correlated positively and strongly with quality of nursing care delivered (*r* = 0.563; *p* = 0.00), with a medium positive correlation with confidence in patients’ and caregivers’ post-discharge management (*r* = 0.357; *p* = 0.00). Overall grade of patient safety also revealed a medium positive correlation with the unit regularly reviewing work processes (*r* = 0.258; *p* = 0.00), staff feeling free to question the actions of those in authority (*r* = 0.222; *p* = 0.00), and the actions of hospital management showing that patient safety is a top priority (*r* = 0.372; *p* = 0.00). Regarding adverse events, overall grade of patient safety showed medium correlations with medication errors (*r* = 0.208; *p* = 0.00) and patient falls (*r* = 0.223 *p* = 0.00). This indicates that, as nurses rated overall patient safety more positively, they would also rate quality of care, confidence in post-discharge management, and positive items on patient safety (AHRQ) better, while at the same time leaning towards a lower incidence of adverse events occurring.

Another strong positive correlation was observed between quality of nursing care, and confidence that patients and their caregivers can manage care after discharge (*r* = 0.438; *p* = 0.00), while medium positive correlations were noted between quality of nursing care and the unit reviewing work processes regularly (*r* = 0.273; *p* = 0.00), staff speaking up if they see something that may negatively impact patient care (*r* = 0.209; *p* = 0.00), staff feeling free to question the actions of those in authority (*r* = 0.210; *p* = 0.00), and the actions of hospital management showing that patient safety is a top priority (*r* = 0.305; *p* = 0.00). Regarding adverse events, quality of nursing care was correlated positively with medication errors (*r* = 0.230; *p* = 0.00), patient falls (*r* = 0.237; *p* = 0.00), and complaints (*r* = 0.249; *p* = 0.00). This shows that the nurses rating the quality of care in their units as more positive would also have more confidence in their patients’ post-discharge management and agree more with positive patient safety items (AHRQ), while indicating a lower incidence of adverse events.

Confidence in post-discharge care and the actions of hospital management showing that patient safety is a top priority were also correlated positively on a medium level (*r* = 0.216; *p* = 0.00). Regarding adverse events, confidence in post-discharge care was correlated positively with patient falls (*r* = 0.202; *p* = 0.00), healthcare-associated infections (*r* = 0.206; *p* = 0.00), and complaints (*r* = 0.211; *p* = 0.00). To explain, this indicates that nurses with a higher rating in confidence in post-discharge management would also have a higher rating of their hospital management’s actions showing that patient safety is a top priority, while also rating the incidence of patient falls, healthcare-associated infections and complaints as occurring less often.

## Discussion

This national study sought to examine nurses' perceptions of the practice environment, quality of care and patient safety across four hospital levels within the public health sector of South Africa. In the participating hospitals we found that there was a negative nurse practice environment, and reports of poor quality of care and patient safety.

The findings on the perceived poor nurse practice environment in the public hospitals is of great concern. The recent South African Human Resources for Health Strategy 2030 advocates for the support of health personnel to deliver quality services [36]. The findings are worrying given that nurses are the backbone of the health system. A negative practice environment contributes to increased staff turnover and mental health challenges due to a stressful environment [37]. The challenges of unavailability of resources are common in public hospitals [38], but when coupled with poor leadership support could worsen the nurse outcomes. The finding on poor participation in hospital affairs indicates a lack of prioritisation of frontline nurses’ voices in hospital affairs; previous studies also found poor involvement of frontline nurses in policy decision making [39, 40]. This could also mean that nurse managers may need empowerment on how to be supportive to staff and in improving prioritisation of frontline nurses’ voices in decision making. The implication of poor involvement of staff in hospital affairs usually includes retaliation, lack of a sense of belonging, and demotivation [41], and it also indicates weak leadership [42]. System level improvements, including relational leadership focused on prioritising staff involvement in decision making, could improve nursing practice environments in these hospitals [43–45]. Nurses’ perceptions of a negative practice environment have been reported in the literature. A study in Menoufia University Hospital, Egypt, reported that about 66.3% of nurses had a poor perception of the work environment [46]. Equally, a rapid review of literature on positive practice environment in the United Kingdom reported that most articles revealed a negative practice environment [47]. Nurse practice environment scholars concur that improving the nurse practice environment saves resources while building a culture of safety [48], and that prudent nurse managers should prioritise creating practice environments that are conducive to providing quality nursing care, as well as that managers should take the lead in impacting the elements of a positive practice environment [49]. In the context of our study, managers could be advocates for resources, nurse inclusion in hospital affairs, and provide strong leadership support.

The finding that almost half of the participating nurses rated the quality of care in their ward as poor is not new. A study in KwaZulu-Natal province in South Africa that explored nurses’ attitudes in providing care to patients revealed that they reported a poor incidence of nursing care to patients and deliberate disregard of essential patient care [50]. The study also noted that nurses attributed poor quality of nursing care to the attitudes of patients’ relatives or patients themselves, including unsupportive management behaviour. Maphumulo and Bhengu (2019) also report on poor quality of care [51]. As mentioned earlier, there are existing national policies to support quality and safety. For example, the Office of Health Standards Compliance set standards on quality in health care; additionally, in 2013 there was a nationwide quality improvement initiative called ideal facility [52]. Such initiatives focused on setting standards to assess each facility regarding compliance with set criteria for quality care in the facilities, started with the PHC clinics [53] and rolled out to hospital level. These results could mean that hospitals are not adhering to the set standards. Major challenges reported in the South African literature associated with poor quality of patient care include poor infrastructure [54, 55], unavailability of medicine [56, 57], shortage of staff, increased workload, shift work and long working hours [58, 59]. These have contributed to an ongoing cycle of high staff turnover [60]. In line with international evidence, it would appear that the practice environment is closely linked to the quality of care [11].

The findings that nurses rated patient safety in their hospitals as poor could be due to their perceived patient safety culture. Evidence suggests that to achieve patient safety, strong leadership and a culture supportive of learning from errors (rather than a punitive approach) are critical ingredients [61]. In this study nurses reported that they experience a punitive reaction when reporting errors, and that they lacked support from their managers, and this is of great concern. Management response to errors is a critical determinant of patient safety culture; positive reactions to reported errors have been cited to encourage health providers to report them and subsequently improve patient care [62]. The findings resonate with recent South African studies that also found a poor patient safety culture in public hospitals [13, 16]. A punitive reaction to reporting of adverse events remains a global challenge. A qualitative study conducted in South Korea on nurses’ experiences with disclosure of patient safety incidents found that nurses often prefer not to report patient safety incidents [63] due to the reaction expected from managers. An integrative review of literature from January 2010 to December 2020 using 31 papers revealed that a non-punitive reaction to patient safety incident reporting could improve patient safety and learning from errors [64]. Such strategies should be adopted in these participating hospitals.

The findings on weekly and daily reported adverse events and complaints resonate with the perceived poor patient safety. The finding that nurses perceived reactions to reported incidents as punitive could mean that more adverse events are not reported, for fear of the management reaction. Adverse events are a critical indicator of patient safety, hence weekly adverse events reported in a hospital should be a major concern. A qualitative study conducted in Palestine on nurses’ experience of the most common medical errors in the intensive care unit and coronary care unit demonstrated that they usually experience events like medication errors, nursing procedure errors, equipment errors, patient monitoring errors, intravenous medication errors, and resuscitation errors [65]. Similarly, in their study in Ghana Alhassan et al. (2019) noted that the type of errors nurses experience were wrong documentation, wrong intravenous fluid, and blood transfusion [66]. Furthermore, a study in Tehran, Iran on the types and causes of medication errors from nurses’ viewpoint indicated that about 64.55% of nurses reportedly made medication errors, and approximately 31.4% nearly experienced medication errors [67]. In the South African context reporting of adverse events remains a challenge, due to similar fear of reporting patient safety incidents, and evidence suggests that health providers often classify adverse events as minor to avoid reporting them [68]. A need to emphasise a just culture in the nursing environment will improve reporting of adverse events, and learning from these events will further reduce occurrences.

We also observed that when looking at comparison of effect sizes across hospitals, larger hospitals most often revealed better practice environments, quality of care and patient safety outcomes, while small district hospitals had the worst. These findings are not uncommon, as a Korean study also confirmed a strong relationship between practice environment and hospital sizes, concluding that the nurse practice environment varies with hospital size [69]. A distinct difference in the hospital categories compared is bed capacity, and complexity of conditions treated in each category, with more complex conditions seen in higher levels of hospitals. In the South African context, hospital categories also influence decisions on allocation and prioritisation of resources among the hospital categories, with more resources given to the larger hospitals [26]. Availability of resources plays a significant role in improving the nursing practice environment [70], and this is a possible contributor to a negative practice environment in small hospitals, since they often have fewer staff and resources [49]. We also found that central hospitals reported more confidence in patients’ ability to manage care after discharge than the smaller hospitals did; this could mean that central hospitals have prioritised teaching of their patients, thereby empowering them for post-discharge care. A 2020 study in the United Kingdom also reported that hospital size is a good predictor of efficient discharge processes [71].

The finding that patient safety in central hospitals was better than in small district hospitals is contrary to the assumption that large hospitals are busy and likely to be attending to complex patient conditions which could make them more prone to errors and unsafe practice [72]. In our study better safety in tertiary hospitals could be related to the fact that they are operated mostly by specialised health professionals who may be more knowledgeable than those in non-specialised hospitals, and that tertiary hospitals are teaching hospitals, often with ongoing training related to practice [26, 73]. In the South African context to our knowledge this is the first study to link nurse practice environment, quality of care and patient safety in four hospital levels, and showing definite differences in nurses’ perceptions at these different levels of care. It followed a quantitative approach, and it would be interesting to further explore the reasons for the perceived practice environment, quality of care and patient safety using qualitative approaches in these hospitals. The findings of this study, specifically the variations in perceived nurse practice environment, quality of care and patient safety across hospital levels, imply an urgent need for mindfulness in resource allocation so as not to compromise care in the smaller hospitals.

The finding of strong correlations between the nurse practice environment, quality of care and patient safety is similar to those of other studies that also emphasised that a negative practice environment is associated with perceived poor quality of care and patient safety [48, 49, 74, 75]. For our study it implies a need to intentionally improve the nurse practice environment, in order to influence quality of care and patient safety. It also means that quality of care and patient safety policies should deliberately consider the practice environment. This could start by involving nurses in policy development, so they can contribute to hospital affairs and the feasibility of the policies. This will also make them feel included as important role-players in the health system. Patient safety policies could also not focus on reporting of errors but consider system level contributors to errors; such practice will also address the reported challenges of punitive reactions to reported errors.

### Limitations and strengths

Since this was a cross-sectional, self-reported survey, one of the limitations could be that the nurses may have had social-desirable bias in their responses, although the authors did try to control for this by asking neutrally worded questions, using anonymous surveys, ensuring that the answer options were not leading and that the order of the answers was randomised. There are several strengths of this study: firstly a contribution to knowledge of a link between nurse practice environment, quality in health care and patient safety in the South African context; often studies investigating these concepts are isolated. An additional strength is that we included a large sample size, representing all nine provinces of South Africa. To our knowledge this is the first study examining nurses' perceptions of the practice environment, quality of care and patient safety across four hospital levels within the public health sector of South Africa.

### Recommendations

There are several recommendations from this study which could contribute to improvement to nurse practice environment, quality of care and patient safety. For example, there is a need to improve organisational culture with a focus on empowering leaders on leading compliance to existing policies, and on supportive leadership; this would lead to an improved nurse practice environment, quality of care and patient safety. A specific focus should be placed on support and empowerment of nurses working in more rural and smaller hospitals. Resource allocation to smaller hospitals should be reviewed, considering the added expenditure associated with remote locations and the added challenges in achieving economies of scale. Enabling positive nursing practice environments by means of enhanced nurse participation, non-punitive strategies of enhancing quality of care, leadership championing and better resource auditing will create environments nurses can thrive in, while also maximising patient outcomes in terms of quality of care and patient safety. In addition, there is an urgent need to review existing policies to identify how the nurse practice environment is enhanced or negatively affected by such policies, and intentionally examine and improve the link between nurse practice environment, quality of care and patient safety in the existing policies.

## Conclusion

Nurses perceived the practice environment, quality of care and patient safety to be poor across four hospital levels within the public health sector of South Africa. Since there is a strong correlation between nurse practice environment, quality of care and patient safety, there is a need to review the existing policies on quality of care and patient safety and if and to what extent they enhance the nursing practice environment. In addition, strengthening compliance with existing policies that enhance quality of care and patient safety remains important, including the creation of a culture that supports a positive nurse practice environment characterised by manager support, nurse participation in hospital affairs and increased supply of resources, especially in smaller and more rural hospital settings.

## Data Availability

The datasets used and/or analysed during the current study are available from the corresponding author on reasonable request.

## Abbreviations

HSOPSC: Hospital Survey on Patient Safety Culture
NDoH: National Department of Health
PES-NWI-R: Practice Environment Scale of the Nurse Work Index Revised
PHC: Primary healthcare
UHC: Universal Health Coverage
